# Digital health data-driven approaches to understand human behavior

**DOI:** 10.1038/s41386-020-0761-5

**Published:** 2020-07-12

**Authors:** Lisa A. Marsch

**Affiliations:** Center for Technology and Behavioral Health, Geisel School of Medicine, Lebanon, NH USA

**Keywords:** Biomarkers, Risk factors

## Abstract

Advances in digital technologies and data analytics have created unparalleled opportunities to assess and modify health behavior and thus accelerate the ability of science to understand and contribute to improved health behavior and health outcomes. Digital health data capture the richness and granularity of individuals’ behavior, the confluence of factors that impact behavior in the moment, and the within-individual evolution of behavior over time. These data may contribute to discovery science by revealing digital markers of health/risk behavior as well as translational science by informing personalized and timely models of intervention delivery. And they may help inform diagnostic classification of clinically problematic behavior and the clinical trajectories of diagnosable disorders over time. This manuscript provides a review of the state of the science of digital health data-driven approaches to understanding human behavior. It reviews methods of digital health assessment and sources of digital health data. It provides a synthesis of the scientific literature evaluating how digitally derived empirical data can inform our understanding of health behavior, with a particular focus on understanding the assessment, diagnosis and clinical trajectories of psychiatric disorders. And, it concludes with a discussion of future directions and timely opportunities in this line of research and its clinical application.

## Introduction

### Overview and limitations of theoretical models of human behavior and diagnostic models of psychiatric disorders

Human behavior is one of the biggest drivers of health and wellness as well as mortality and morbidity. Indeed, health risk behavior, including poor diet, physical inactivity, tobacco, alcohol, and other substance use, causes as much as 40% of the illness, suffering, and early death related to chronic diseases [1–3]. Health risk behavior is linked to obesity, Type 2 diabetes [4], heart disease, liver disease, kidney failure, and neurological diseases. It is also linked to many mental health disorders including anxiety and depression [5, 6]. And it greatly increases one’s risk for a wide variety of cancers. For example, heavy alcohol use greatly increases risk of breast [7–9], esophageal, and upper [10] digestive and liver cancers [11, 12]. Smoking is strongly linked to lung cancer and is also a major contributor to esophageal cancer [13–16]. And, obesity increases risk of colorectal and esophageal cancer [17–19].

Research designed to explain and predict health behavior and events influencing health outcomes has heavily relied on theoretical models of health behavior and behavior change [20, 21]. At the psychological level, the cognitive literature has focused on such performance-related processes as goal maintenance in working memory, impulsivity, and cognitive homeostasis. The affective science and social psychology literatures have focused on emotion regulation processes, social influences and resource models. In parallel, the health psychology and behavioral medicine literatures have focused on processes, such as self-efficacy and outcome expectancies. At the behavioral level, focus has been largely placed on behavioral disinhibition and temporal discounting. At the neural level [22], health behavior can be conceptualized in terms of top-down control (implemented by fronto-parietal networks) over impulsive drives or habits (implemented by subcortical and ventromedial prefrontal regions [23]). And an emerging framework from neuroeconomics has characterized decision processes in terms of goal-directed versus habitual or Pavlovian control over action [24–26].

Overall, these models afford a conceptual framework for illustrating causal processes of key constructs hypothesized to influence or change a target behavior. Theoretical models may be useful for developing, implementing, and evaluating behavior change interventions. And, interventions informed by theories of human behavior are generally more effective compared with those that are not [27]. Collectively, various theoretical models have articulated that an individual’s beliefs and attitudes, behavior intentions, level of motivation for behavior change, and social and cognitive processes impact health behavior [28].

Despite the promise of theoretical models of health behavior, their ability to explain and predict health behavior has been only modestly successful [28–30]. Many theoretical models have regarded human behavior as linear or static in nature and have not recognized that behavior is dynamic and responsive to diverse social, biological, and environmental contexts. And, theoretical models have heavily focused on between-person differences in behavior and have not embraced the study of important within-person differences in behavior. Further, many theoretical models of health behavior and behavior change have often been derived within siloed disciplines (e.g., health psychology, neuroscience) with little crosstalk [31, 32].

In addition, research examining factors that influence health behavior has tended to examine a small set of potential moderators or mediators of health behavior at a specific level of analysis (e.g., emotion regulation alone or impulsivity alone) and may lead to over-simplified accounts of behavior [33–36] change. Finally, little research has established the temporal precedence of a broad array of potential factors impacting health behavior [37]. More frequent and longer assessment of moderators, mediators, and outcome(s) will be necessary to elucidate the temporal dynamics between changes in specific mechanisms [38] and behavior [39, 40].

Similar limitations are evident in our current models for understanding and determining clinical diagnoses for psychological or psychiatric disorders. The current process for identifying diagnosable disorders heavily relies on measuring the number and type of symptoms that a person may be experiencing as well as associated distress or impairment. Although this current diagnostic process provides a useful common language of mental disorders for clinicians, the process is largely based on consensus from expert panels and may oversimplify our understanding of human behavior [41]. And indeed, many mental health clinicians do not measure behavior, cognition, and emotion when ascribing a psychiatric disorder to a patient. Further, mental health professionals usually interact with, and provide diagnoses to, patients at a specific moment in patients’ lives, but recent evidence shows that people with psychological disorders may experience many different kinds of disorders across diagnostics families over their lifespan [42]. There is tremendous opportunity to understand psychological/biological systems that span the full range of human behavior from normal to abnormal and to empirically assess how they are situated in environmental and neurodevelopmental contexts [43]. Examining a broad array of factors impacting health behavior at multiple levels of empirical analysis and over time will enable a more comprehensive picture of health behavior and will increase our ability to develop more impactful interventions and better understand the conditions under which replications of effects do and do not occur.

## The promise of digital health in understanding human behavior

Advances in digital technologies and data analytics have created unparalleled opportunities to assess and modify health behavior and thus accelerate the ability of science to understand and contribute to improved health behavior and health outcomes. Digital health refers to the use of data captured via digital technology to measure individuals’ health behavior in daily life and to provide digital therapeutic tools accessible anytime and anywhere [44, 45]. For example, smartphones have an array of native sensors including Bluetooth, GPS, light sensor, accelerometer, microphone, and proximity sensors as well as systems logs of calls, and short message service use. Smartphones, as well as some wearable devices (e.g., smartwatches), thus enable passive, ecological sensing of behavioral, and physiological features, such as one’s sleep, physical activity, social interactions, electrodermal activity, and cardiac activity [46]. Individuals can also offer responses to questions they are prompted to answer on mobile devices (sometimes called “ecological momentary assessment” or EMA) to provide snapshots into, for example, their context, social interactions, stress, pain, mood, eating, physical activity, mental health symptoms, and substance use. And, social media data, that many individuals produce in high volume, provide information about individuals’ behavior, preferences, and social networks. These “digital exhaust” [47] data or “digital footprints” [48] enable the continuous measurement of individuals’ behavior and physiology in naturalistic settings.

These digital data may greatly complement and extend traditional sources of clinical data (which is typically captured on an episodic basis in a clinical context) with intensive, longitudinal ecologically valid data. Digital health data capture the richness and granularity of individuals’ behavior, the confluence of factors that impact behavior in the moment, and the within-individual evolution of behavior over time. As such, they may contribute to discovery science by revealing digital markers of health and risk behavior [49, 50]. They may help us to better develop empirically based diagnostic classifications of aberrant/dysfunctional behavior and the clinical trajectories of diagnosable disorders over time [50]. And, they may help us in translational science by informing more personalized, biomarker-informed, and timely models of intervention delivery.

As the majority of the world has access to digital technology—indeed, there are 8 billion mobile phone subscriptions worldwide [51]—digital health data-driven approaches can be used to understand human behavior across the population.

## The state of the science of digital health data-driven approaches to understand human behavior

This manuscript provides a review of the state of the science of digital health data-driven approaches to understanding human behavior. The manuscript first describes various methods of digital health assessment and sources of digital health data. It then provides a synthesis of the scientific literature evaluating how digitally derived empirical data can inform our understanding of health and risk behavior. It then focuses on how digital health may help us to develop a better empirically based understanding in the assessment, diagnosis, and measurement of clinical trajectories of aberrant/dysfunctional disorders in the field of psychiatry (a field that has led pioneering research in digital health [52]). Finally, it concludes with a discussion of future directions and timely opportunities in this line of research and its clinical application, including the development of personalized digital interventions (e.g., behavior change interventions) informed by digital health assessment.

### Digital health assessment methods

Although digitally derived data have been used to understand behavior and context in the field of Computer Science for over 15 years, a primary term currently used to capture digital health assessment is “digital phenotyping” [53] and is increasingly used by scientists, funders, as well as the popular press. Digital phenotyping [54] primarily employs passively sensed data to allow for a moment-by-moment (in situ) quantification of behavior. These data can include data derived from smartphone or smartwatch sensors (e.g., an individual’s activity, location), features of voice and speech data collected by mobile devices (e.g., prosody and sentiment), as well as data that captures a person’s interaction with their mobile device (e.g., patterns of typing or scrolling). Digital phenotyping largely employs passive data (to reduce burden to participants in data collection), and some researchers confine their definition of digital phenotyping to passive data. However, digital measurement and analytics also encompass many other sources of data that are actively generated by individuals, including social media data, EMA data, and online search engine activity.

Overall, digital phenotyping focuses on the use of such digital data to understand and predict health outcomes of behaviors of interest. Sophisticated inferences from these data are increasingly possible due to the rapidly advancing fields of big-data analytics and advanced Artificial Intelligence (including advanced machine learning approaches that focus on the creation of systems that learn from data instead of simply following programmed instructions).

Behavioral health systems that leverage passive sensing and machine learning to learn and adapt to a person’s actual behavior and surroundings offer a promising foundation for predictive modeling of an individual’s behavioral health trajectory and may support new breakthrough intervention technologies targeting health behavior. These developments enable behavioral monitoring to occur in the background as individuals go about their lives and build dynamic computational models tailored to the user that can lead to effective interventions.

And digital phenotyping may reveal new insights into how other data sources (such as genetic, molecular and neural circuitry data) interrelate with clinically observable psychopathology [55, 56].

### Overview of the scientific literature on the application of digitally derived empirical data to understand health behavior and psychopathology

A robust and rapidly growing scientific literature is increasingly demonstrating the potential utility of digital assessment in revealing new insights into human behavior, including psychological and psychiatric disorders.

#### Digital health biomarkers of health and risk behavior captured via mobile technology

Continuous smartphone sensing (e.g., of activity, mobility, sleep) has been shown to be significantly linked to mental well-being, academic performance (Grade Point Average), and behavioral trends of a college student body, such as increased stress, reduced sleep, and reduced affect as the college term progresses and stress increases [57]. These patterns may help us to understand, in close to real time, when individuals may be at risk of academic and/or mental health decline. Assessment of individual’s interactions with mobile devices (e.g., swipes, taps and keystroke events) have been shown to capture neurocognitive function in the real world and may provide an ecological surrogate for laboratory-based neuropsychological assessment [58]. And, continuous smartphone monitoring can measure brain health and cognitive impairment in daily life [59]. And, digital data derived from mobile sensing (e.g., calling, texting, conversation and app use) have also been used to characterize behavioral sociability patterns and to map these behaviors onto personality traits [60]. Further, phenotypic data gathered via wearable sensors have shown that several metrics of sleep (total sleep time and sleep efficiency) are associated with cardiovascular disease risk markers, such as waist circumference and [61] body mass index and that insufficient sleep is linked to premature telomere attrition. Thus, these digitally derived health risk data can provide real time insights into biological aging.

#### Digital health measurement of aberrant/dysfunctional behavior and the clinical trajectories of diagnosable disorders over time captured via mobile technology

Digital assessment has also illuminated novel insights into the nature and course of psychological and psychiatric disorders. High-frequency assessment of cognition and mood via wearable devices among persons with major depressive disorder has been shown to be feasible and valid over an extended period [62]. Behavioral indicators passively collected through a mobile sensing platform (e.g., the sum of outgoing calls, a count of unique numbers texted, the dynamic variation of voice, speaking rate) have been shown to predict symptoms of depression and PTSD [63]. Features derived from GPS data collected via phone sensors, including location variance, entropy, and circadian movement, have been shown to predict severity of depressive symptoms and that these relationships can differ at different points in time (e.g., weekend vs. weekday [64]). And assessment of voice data has identified vocal acoustic biomarkers that have shown promise in predicting treatment response among persons with depression [65].

Movement data from actigraphs alone, a single measure of gross motor activity from a sensor worn on the wrist, were able to identify the diagnostic group status of individuals with major depression or bipolar vs. healthy controls 89% of the time. This level of accuracy in diagnostic classification is greater than published inter-reliability rates for second raters using the Structured Clinical Interview for the DSM (SCID). And results showed that actigraphy data predicted the majority of variation in patients’ depression severity over an ~2-week period [66].

Emotion dynamics captured over time via digital technology have been shown to differentially predict bipolar and depressive symptoms concurrently and prospectively [67]. And, EMA data captured on smartphones has been shown to predict future mood among persons with bipolar disorder [68]. In addition, smartphone usage patterns have been shown to be linked to functional brain activity related to depression. For example, phone unlock duration has been shown to be positively linked to resting-state functional connectivity between the subgenual cingulate cortex (an area understood to be involved in depression) and the ventromedial/orbitofrontal cortex [69]. Results suggest that digital biomarker data may reflect readily capturable data that relate to brain functioning.

Further, a small pilot study evaluated changes in mobility patterns and social behavior among persons diagnosed with schizophrenia using passively collected smartphone data. Results indicated that the rate of behavioral anomalies that were identified in the 2 weeks prior to a clinical relapse were markedly higher (71%) than rates of behavioral anomalies during other periods of time [70]. And, other research has underscored the significant variability across individuals in digital indicators of a psychotic relapse [71] thus underscoring the multi-dimensional nature of a diagnosis of a psychotic disorder.

In addition, a small series of case studies demonstrated that self-reported psychotic symptoms are linked to various behaviors (cognition scores on games) and activity levels (step count) among persons with psychotic illness. Importantly, results revealed considerable variability in the patterns in these data streams across individuals, underscoring the utility of these approaches in understanding and monitoring within-individual clinical trajectories [72]. And other research has demonstrated that decreased variability in physical activity and noisy conditions on an inpatient psychiatric unit, captured via multimodal measurement, is associated with violent ideation among inpatients with serious mental illness [73].

Assessment of geography via passive sensing of geolocation using GPS has demonstrated that drug craving, stress, and mood among persons with an opioid use disorder were predicted by exposure to visible signs of environmental disorder along a GPS-derived [74, 75] track (such as visible signs of poverty, violence, and drug activity). A recent digital health EMA study demonstrated a stronger link between drug craving and drug use than between stress and drug use—a result that was not well-documented or understood from prior traditional clinical assessment [76]. And, among smokers trying to quit, lapses to smoking were shown to be associated with increases in negative mood for many days (and not just hours) before a smoking lapse [77]. These studies reveal new insights into the dynamic nature of drug use events and the confluence of factors that impact them.

Unfortunately, only a few studies have included randomized controlled evaluations of the clinical utility of digital phenotyping in the clinical treatment of psychological disorders. Among these studies, one recent, controlled study that investigated the effect of smartphone monitoring of persons with bipolar disorder did not show a statistically significant benefit on depressive or manic symptoms compared with a control group, although persons with smartphone monitoring reported higher quality of life and lower stress [78, 79].

#### Digital health measurement of health behavior captured via additional (non-mobile) data sources

In addition to data captured via mobile devices, other sources of digital data have been shown to reveal insights into human behavior. For example, social media data have provided new insights into mental health and substance use behavior. In one study, a deep-learning method was able to identify individuals’ risk for substance use using content from their Instagram profiles [80]. And another evaluation demonstrated that community-generated Instagram data (post captions and comments from friends or followers), when evaluated along with user-generated content (individuals’ post captions and comments), were able to identify depression among individuals. Other work has also demonstrated that Facebook status updates can predict postpartum depression [81] and that depression can be identified via daily variation in word sentiment analysis among Twitter and Facebook users [82, 83]. Such methods offer promise for conducting population-level risk assessments and inform population-level interventions [84].

Data from online search engine activities are another source of consumer-generated digital data that can reveal individual-level as well as population-level behavioral patterns. For example, online health-seeking behavior has been shown to predict real-world healthcare utilization [85]. Online search activity has been shown to be related to changes in use of new substances [86], and substance use search data have been strongly correlated with overdose deaths [87]. And, a recent study analyzed over 10 million Google search queries across the United State related to mental health during the COVID-19 global pandemic. Results revealed that mental health search queries increased rapidly prior to the issuance of stay-at-home order within states, and these searches markedly decreased after the announcement and implementation of these orders, presumably once a response/management plan was in place [88].

## Future research directions/opportunities

Overall, the existing scientific literature demonstrates a compelling “proof of concept” that digital health data can provide new insights into human behavior, including psychopathology. This line of research offers great promise for advancing our theoretical models of health behavior and informing behavior change interventions that are responsive to the dynamic nature of health behavior.

The promise of digital health is particularly compelling when applied to the field of psychiatry. Digital assessment allows for the continuous, empirical quantification of clinically useful digital biomarkers that can be useful in identifying and refining diagnostic processes over time. These data may also be useful as outcomes in measurement-based care. These data may help us to generate predictive models that reflect the confluence of factors, and their relations over time, that may inform when an individual may be at risk for a clinically significant event (such as a relapse or psychotic event). These methods may help detect a problem before it occurs and inform in-the-moment preventative interventions. And, given that psychiatric conditions are often chronic and recurrent, digital data captured in an intensive longitudinal manner can inform strategies for optimizing responsive and adaptive models of clinical care over time.

Thus, digital health offers value along a full spectrum from measurement to intervention delivery—by providing novel digital biomarkers, new insights into clinical diagnoses of psychiatric disorders, personalized intervention delivery on digital platforms, as well as digital outcome measurement over time. These multiple applications of digital health can complement one another by measuring behavior and informing interventions that are responsive to that measurement.

Despite the promise of digital health data-driven approaches to understanding human behavior, there remain many gaps and opportunities in the field. As noted above, most digital health research has not embraced rigorous experimental research designs. Indeed, only a paucity of trials has embraced well-powered, randomized, controlled research designs to allow for causal inference about the value of digital assessment and associated data analytics in informing clinical outcomes [89]. In addition, tremendous variability exists in the specific digital metrics being employed in digital health research—ranging from smartphone sensing data, smartwatch sensing data, EMA data, social media data, and online search engine data. And within each of these categories, there is also great variability in the types of features that are being extracted and applied to clinical inference. For example, in smartphone sensing alone, some research focuses heavily on GPS, other work focuses on actigraphy, while still other research focuses on movement. The specific features and sources of digital health data (including the potential combination of multiple sources of digital data) that provide maximal precision in characterizing human behavior and behavioral disorders remain understudied as do the psychometric characteristics (e.g., validity and reliability) of such metrics [90]. In order to realize the potential of digital health and provide the most robust and replicable results, a priority focus on experimental rigor and reproducibility is critically needed.

In addition, digital health research to date has been conducted within our existing classification systems (e.g., patients with bipolar disorder or depression) which, as noted above, can be refined with digital health approaches. And most digital health research has been focused on disease-specific models (e.g., focusing on depression alone or substance use alone). The rich, granular data afforded by digital health approaches offer tremendous opportunity to transcend siloed disease-specific models of behavior and care to empirically embrace, understand, and treat the complexity and interrelatedness of behavioral patterns and clinical disorders. Indeed, scientific research has demonstrated that many disorders co-occur and interrelate in meaningful ways and that these disorders evolve and change over the lifespan. Digital health offers great (but yet unrealized) promise to provide a data-informed understanding of this full spectrum of health and wellness. This may include the development of an ontology of behavior that is informed by digital health data, which may enable a new understanding of co-occurring aberrant/dysfunctional behaviors and their evolution over time. And this may include digital therapeutic interventions that are responsive to the combination of needs and goals of each individual and their evolution over time.

Finally, much of the current research appears to ground in assumptions that digital health data will be of interest and of value to consumers, patients, and clinicians. Although one could make the case that patients may value self-monitoring and feedback on their behavior and their clinical status and that clinicians may welcome actionable digital health data that can aid them in the care of patients, this may not always be the case. For example, if patients do not experience value in generating and sharing these data, they will not be inclined to do so (or to do so for any extended period of time). If providers receive large volumes of unsolicited data and/or data that do not directly inform their clinical work, they may perceive such a model to be burdensome and unhelpful. And if patients do not understand the privacy and security considerations of how their sensitive data will be handled and/or if healthcare systems do not understand data sharing/protection policies of industry vendors, this will undoubtedly impact adoption [91]. Indeed, it is possible that the current scientific literature largely reflects a subset of the population that are willing to share personal health data collected on digital devices, which may not be broadly generalizable. A broader dialog is needed to establish fundamental principles of privacy and research ethics in the digital health space. This may include establishing best practices for ensuring protections of patient privacy and sensitive information while still allowing for data to be shared between parties (e.g., patients and clinicians) in accordance with patient and provider preferences. And, this may include informed consent processes that are adaptive and dynamic in response to each individual’s digital literacy and data sharing preferences [92]. Overall, as research and clinical application of digital measurement of behavior expands, there is an urgent need to ensure that implementation science approaches are employed to systematically assess the preferences of all the relevant digital health stakeholders and to inform models of development and deployment that have the greatest chance of scalability and sustainability. This will undoubtedly require an interdisciplinary effort across the scientific arena (including behavioral science, data science, computer science and neuroscience) as well as the digital health industry and experts in public policy.

## Conclusion

Digital health and data analytics are transforming our world. And, the real-world precision assessment that digital health methods enable are providing unprecedented insights into human behavior and psychiatric disorders and can inform interventions that are personalizable and adaptive to individuals’ changing needs and preferences over time. Now is the moment of opportunity to embrace a systematic, rigorous, and comprehensive research agenda to realize this vision.

## Funding and disclosure

Research reported in this publication was supported by the National Institute on Drug Abuse of the National Institutes of Health [Grant number P30DA029926]. The author is affiliated with Pear Therapeutics, Inc., HealthSim, LLC, and Square2 Systems, Inc. Conflicts of interest are extensively managed by her academic institution, Dartmouth College.
